# Targeting Lysyl Oxidase-like 2: A Therapeutic Strategy for Idiopathic Pulmonary Fibrosis with a Novel Indolizine Derivative

**DOI:** 10.3390/pharmaceutics18050554

**Published:** 2026-04-30

**Authors:** Doo Hee Shim, Min Jung Kim, Hyeon Woo Chung, Mi Na Kim, Myung Hyun Sohn, Sunhee Lee, Ikyon Kim, Chun Geun Lee, Jack A. Elias, Jeon Han Park, Jae Myun Lee

**Affiliations:** 1 Department of Microbiology and Immunology, Institute for Immunology and Immunological Diseases, Brain Korea 21 PLUS Project for Medical Science, Yonsei University College of Medicine, Seoul 03722, Republic of Korea; 2 Department of Pediatrics, Yongin Severance Hospital, Yonsei University College of Medicine, Yongin-si 16995, Republic of Korea; 3 Department of Pediatrics, Institute of Allergy, Severance Hospital, Brain Korea 21 PLUS Project for Medical Science, Yonsei University College of Medicine, Seoul 03722, Republic of Korea; 4 College of Pharmacy and Yonsei Institute of Pharmaceutical Sciences, Yonsei University, Incheon 21983, Republic of Korea; 5 Intercollege, Hanyang University, Seoul 04763, Republic of Korea; 6 Department of Molecular Microbiology and Immunology, Brown University, Providence, RI 02903, USA

**Keywords:** idiopathic pulmonary fibrosis (IPF), pulmonary fibrosis, lysyl oxidase-like 2 (LOXL2), extracellular matrix (ECM), indolizine derivative, TGF-β signaling, collagen cross-linking, antifibrotic therapy

## Abstract

**Background:** Idiopathic pulmonary fibrosis (IPF) is a progressive lung disease marked by excessive extracellular matrix (ECM) deposition. Current FDA-approved therapies, such as pirfenidone and nintedanib, offer limited efficacy in halting disease progression. Lysyl oxidase-like 2 (LOXL2) is a key enzyme involved in ECM remodeling and fibrosis. This study investigates Compound **#765**, a novel indolizine derivative, as a potential LOXL2 inhibitor for IPF treatment. **Methods:** Compound **#765** was synthesized and characterized using spectroscopic methods. Its inhibitory effect on LOXL2 activity was evaluated using LOXL2 enzymatic assays, in vitro fibrosis models with human lung fibroblasts, and in vivo models of pulmonary fibrosis, including bleomycin-treated and TGF-β1-overexpressing transgenic mice. In silico docking studies predicted the binding affinity of Compound **#765** to LOXL2. **Results:** Compound **#765** targeted LOXL2 activity and reduced collagen production in lung fibroblasts. In both bleomycin-induced pulmonary fibrosis and TGF-β1-overexpressing murine models, Compound **#765** significantly alleviated fibrosis, as indicated by reduced collagen accumulation and inflammatory cell infiltration. The in silico docking studies predicted favorable binding affinity to LOXL2, which was confirmed through in vitro experiments. Importantly, Compound **#765** suppressed fibrosis-associated markers in fibroblasts derived from IPF patients, suggesting translational potential. **Conclusions:** These results demonstrate that Compound **#765** functions as a LOXL2 inhibitor with significant anti-fibrotic effects in vitro and in vivo, offering a promising therapeutic approach for IPF and other fibrotic lung diseases.

## 1. Introduction

Fibrosis is a prominent pathological feature of chronic injuries and various inflammatory diseases, characterized by the excessive accumulation of extracellular matrix (ECM) components, such as collagen and fibronectin. This ultimately leads to permanent scarring, organ failure, and in advanced stages, fatal conditions such as idiopathic pulmonary fibrosis (IPF), liver fibrosis, and heart failure [1,2].

IPF is a well-recognized prototype of pulmonary fibrosis and is a devastating disease with a median survival of only 3 years [3]. Currently, the U.S. Food and Drug Administration has approved two primary treatments: pirfenidone (Esbriet, InterMune Inc., Brisbane, CA, USA) and nintedanib (Ofev, Boehringer Ingelheim, Ingelheim am Rhein, Germany) [4,5]. While pirfenidone shows significant anti-inflammatory and antifibrotic effects [6,7] and nintedanib, a multi-tyrosine kinase inhibitor, helps slow disease progression [8,9], both drugs have significant limitations. These include low bioavailability, a narrow therapeutic window, and potential liver toxicity, restricting their suitability for all IPF patients [10]. According to recent international guidelines, effective therapeutic options for IPF remain severely limited, underscoring the critical unmet need for safer and more broadly applicable therapies [11].

Lysyl oxidase (LOX) and its four homologs (LOXL1–4) are essential enzymes that catalyze the initial step of collagen and elastin covalent cross-linking during ECM synthesis [12]. Among these, LOXL2 is highly expressed in the fibrotic foci of IPF patients, and its elevated serum levels correlate strongly with disease progression [13,14]. Mechanistically, LOXL2 catalyzes the formation of these stable ECM cross-links, which leads to tissue stiffening. This LOXL2-driven ECM stiffening further enhances fibroblast activation, amplifies TGF-β-mediated profibrotic signaling, and promotes epithelial–mesenchymal transition (EMT) [1]. Through its multifaceted roles in ECM remodeling and signaling, LOXL2 functions as a key driver of irreversible fibrotic remodeling, making it a compelling and actively pursued therapeutic target [15,16,17,18].

Previous clinical efforts to target LOXL2, including antibody-based approaches such as simtuzumab, have yielded limited success, highlighting the challenges associated with effective LOXL2 inhibition in fibrotic tissues. These outcomes suggest that alternative therapeutic modalities may be required to fully exploit LOXL2 as an antifibrotic target [19,20].

Indolizine, a heteroaromatic ring system, has attracted increasing attention in pharmaceutical development due to its favorable drug-like properties and widespread biological activities, including anti-cancer, antiviral, and anti-inflammatory effects [21,22]. Notably, indolizine derivatives have been reported to exhibit anti-inflammatory activity, including modulating key inflammatory mediators such as COX-2, TNF-α, and IL-6 [23]. In fibrotic diseases, inflammatory responses are closely linked to the activation of profibrotic pathways, particularly those mediated by transforming growth factor-β (TGF-β), which drives fibroblast activation and ECM deposition. Therefore, the indolizine scaffold’s anti-inflammatory properties may contribute to modulating fibrosis-related pathways, supporting its potential as a candidate for antifibrotic therapy.

In this context of urgent therapeutic need and broad pharmacological interest, we undertook a long-term screening effort in which thousands of indolizine derivatives were synthesized and tested. Compound **#765** was prioritized because its indolizine-based scaffold demonstrated strong inhibitory activity against LOXL2 with a favorable preliminary safety profile. We then comprehensively assessed the inhibitory effect of this compound on LOXL2 enzymatic activity and its impact on fibrosis progression both in vitro and in vivo, establishing a mechanistic link between LOXL2 targeting and anti-fibrotic efficacy.

## 2. Materials and Methods

### 2.1. Synthesis and Characterization of Compound #***765***

**Figure 1:**
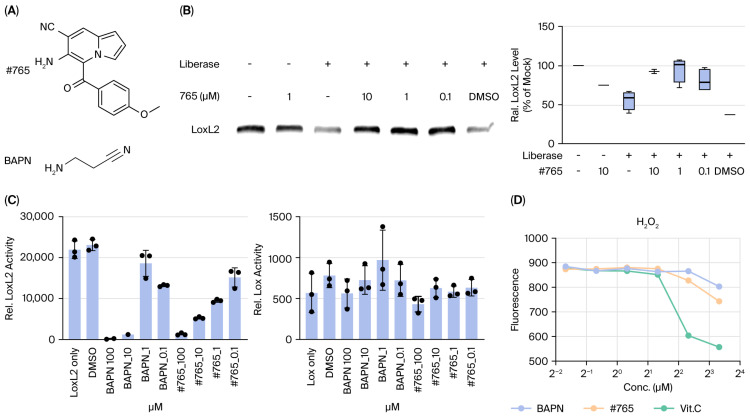
Compound **#765** interacts with lysyl oxidase-like 2 (LOXL2) and inhibits its enzymatic activity. (**A**) Chemical structures of Compound **#765** and the pan-LOX inhibitor β-aminopropionitrile (BAPN). (**B**) Drug Affinity Responsive Target Stability (DARTS) assay showing that LOXL2 is degraded by liberase in the absence of inhibitor, whereas co-incubation with Compound **#765** protects LOXL2 from proteolysis in a dose-dependent manner. Band intensities were quantified using integrated density (ImageJ, version 1.54, NIH, Bethesda, MD, USA) and normalized within each independent experiment. Values were expressed relative to the mock control (set to 100%). In experiments without mock controls, values were normalized to the corresponding liberase-treated condition. (**C**) LOXL2 enzymatic activity assay demonstrating that both BAPN and Compound **#765** inhibit LOXL2 in a dose-dependent manner. Compound **#765** does not inhibit LOX enzymatic activity under identical conditions. LOXL2 activity was quantified using the Amplex Red/Resorufin assay, where H_2_O_2_ produced by LOXL2 is converted to a fluorescent product by peroxidase. (**D**) Hydrogen peroxide assay confirming that Compound **#765** itself does not produce H_2_O_2_, indicating that the decreased fluorescence in (**C**) reflects specific inhibition of LOXL2 catalytic activity rather than interference or background signal. Data are presented as mean ± SD (*n* = 3–5). BAPN, β-aminopropionitrile; LOXL2, lysyl oxidase-like 2; SD, standard deviation.

Compound **#765** (6-Amino-5-(4-methoxybenzoyl)indolizine-7-carbonitrile, Figure 1A) was prepared according to a previously reported method [24]. A solution of 1-(2-(4-methoxyphenyl)-2-oxoethyl)-1*H*-pyrrole-2-carbaldehyde (500 mg, 2.56 mmol) in ethanol (7.5 mL) was treated with piperidinium acetate (0.5 equiv) and malononitrile (1.5 equiv) at room temperature (RT). The mixture was heated at 120 °C for 24 h, concentrated under reduced pressure, and purified by silica gel column chromatography (hexane:ethyl acetate:dichloromethane = 10:1:2) to form Compound **#765** (605.5 mg, 73%) as an orange solid. Melting points were measured using an IA9100 melting point apparatus (Electrothermal, Staffordshire, UK). ^1^H and ^13^C NMR spectra were recorded on a 400 MHz NMR spectrometer (Agilent Technologies, Santa Clara, CA, USA). HRMS were measured using an electrospray ionization (ESI) source on a 6550 iFunnel quadrupole time-of-flight (Q-TOF) mass spectrometer (Agilent Technologies, Santa Clara, CA, USA). Spectroscopic data confirmed the structure. mp: 131.4–132.1 °C (605.5 mg, 73%); ^1^H NMR (400 MHz, CDCl_3_) δ 7.81 (s, 1H), 7.54 (d, *J* = 8.4 Hz, 2H), 7.00 (s, 1H), 6.89 (d, *J* = 8.8 Hz, 2H), 6.71 (d, *J* = 4.4 Hz, 1H), 6.52 (dd, *J* = 4.0, 2.8 Hz, 1H), 5.82 (s, 2H), 3.86 (s, 3H); ^13^C{^1^H} NMR (100 MHz, CDCl_3_) δ 188.3, 163.3, 139.2, 130.8, 130.7, 129.9, 128.2, 122.5, 116.6, 114.4, 114.3, 113.3, 108.0, 93.5, 55.5; HRMS (ESI-QTOF) *m*/*z* [M + H]^+^ calcd. for C_17_H_14_N_3_O_2_ 292.1081, found 292.1085. Purity was determined to be 99.4% by HPLC analysis (λ = 254 nm). All analytical data are provided in Supplementary Figures S1–S3. Stock solutions of Compound **#765** (10 mM) were prepared in dimethyl sulfoxide (DMSO) and stored at −20 °C. The final concentration of DMSO in all in vitro assays did not exceed 0.1% (*v*/*v*).

### 2.2. Drug Affinity Responsive Target Stability (DARTS) Assay

The DARTS assay was performed with minor modifications to a previously established method [25]. Recombinant human LOXL2 protein (50 ng, Cat#2639-AO; R&D Systems, Minneapolis, MN, USA) was incubated with Compound **#765** in proteolysis reaction buffer (50 mM Tris-HCl [pH 8.0], 50 mM NaCl, and 10 mM CaCl_2_) for 120 min at 9 °C. Proteolysis was initiated by adding Liberase TM protease (1 μg, Cat#5401119001; Sigma-Aldrich, St. Louis, MO, USA) and incubating for 20 min at 37 °C. An equivalent volume of DMSO was used as a negative control. Reactions were terminated on ice, and stability was analyzed by SDS-PAGE followed by Western blotting using an anti-LOXL2 antibody (cat #ab96233; Abcam, Cambridge, UK).

### 2.3. LOXL2 Activity and Hydrogen Peroxide Scavenging Assay

LOXL2 (25 ng; R&D Systems) and LOX (50 ng, Clone#RPC580Hu01; Cloud-Clone Corp., Katy, TX, USA) proteins were incubated with Compound **#765** for 1 h at RT. Lysyl oxidase activity was measured using the Lysyl Oxidase Activity Assay Kit (Cat#ab112139; Abcam) according to the manufacturer’s instructions. Hydrogen peroxide levels were measured using the Amplex Red Hydrogen Peroxide/Peroxidase Assay Kit (Cat#A22188; Thermo Fisher Scientific, Waltham, MA, USA) in the absence of LOXL2. The inhibitory effects of Compound **#765**, β-aminopropionitrile (BAPN), and ascorbic acid were evaluated by measuring fluorescence changes with background fluorescence (buffer or drug only) subtracted.

### 2.4. Cell Culture and Treatment

Human fetal lung fibroblasts (MRC5; ATCC, Manassas, VA, USA) were cultured in Minimum Essential Medium (MEM; Hyclone, Logan, UT, USA) supplemented with 10% fetal bovine serum (FBS; Hyclone) and 1% penicillin/streptomycin (P/S; Hyclone). Primary human lung fibroblasts (LL24 and LL97A; ATCC) were cultured in Kaighn’s Modification of Ham’s F-12 Medium (F-12K; ATCC) with 15% FBS (Hyclone) and 1% P/S. Cells were maintained at 37 °C in a 5% CO_2_ incubator. For the experiments, cells between passages 3 and 18 were used. To induce serum starvation, cells were incubated in medium containing 0.5% FBS for 24 h, then were treated with recombinant human TGF-β1 (2.5–5 ng/mL; R&D Systems) and Compound **#765**. Cells were harvested at 24, 48, and 72 h for protein expression, collagen, and qPCR.

### 2.5. Immunofluorescence Assay

Cells were fixed with 4% paraformaldehyde for 15 min at RT, permeabilized with 0.4% Triton X-100 in PBS for 10 min, and subsequently blocked with 2% bovine serum albumin (BSA) in PBS for 1 h at RT. Primary antibodies were applied overnight at 4 °C at the manufacturer-recommended dilutions: anti-collagen I (Cat#72026; Cell Signaling Technology, Danvers, MA, USA), anti-alpha-smooth muscle actin (α-SMA; Cat#A2547; Sigma-Aldrich), and anti-fibronectin (FN; Cat#ab2413; Abcam). Afterward, Alexa Fluor conjugated secondary antibodies (Cat#A-11008; Invitrogen, Carlsbad, CA, USA) for 1 h at RT. Nuclei were counterstained with Hoechst 33342 (Cat#R337605; Invitrogen) for 15 min at RT. Samples were analyzed using a Leica THUNDER Imaging System operated with LAS X software (Leica application suite X 3.7.4.23469, Leica Microsystems, Wetzlar, Germany).

### 2.6. Western Blotting

Cells were lysed with RIPA buffer (Thermo Fisher Scientific) containing a Halt protease inhibitor cocktail (Thermo Fisher Scientific). Protein concentrations were determined using the Pierce BCA Protein Assay Kit (Thermo Fisher Scientific). Lysates were separated on NuPAGE SDS-PAGE gels (Novex, San Diego, CA, USA), transferred to nitrocellulose membranes (Invitrogen), and blocked with 3% BSA in TBS-T for 1 h at RT. The membranes were incubated overnight at 4 °C with primary antibodies: anti-LOXL2 (Cat# ab96233, Abcam), anti-α-SMA (Sigma-Aldrich), and anti-FN (Abcam) followed by horseradish peroxidase (HRP)-conjugated secondary antibodies (Cell Signaling Technology) for 1 h at RT. Bands were visualized using the WesternBright ECL detection reagent (Advansta, Menlo Park, CA, USA) and imaged using an ImageQuant LAS 4000 system (GE Healthcare Bio-Sciences AB, Uppsala, Sweden).

### 2.7. RNA Isolation and qRT-PCR

Total RNA was extracted using TRIzol reagent (Invitrogen). qRT-PCR was performed with the Luna Universal Probe One-Step RT-qPCR Kit (New England Biolabs, Ipswich, MA, USA) on a QuantStudio3 Real-Time PCR System (Applied Biosystems, Carlsbad, CA, USA). For TGF-β1, α-SMA, PDGFR-β, VEGF, and β-actin, the following primer sets were used: TGF-β1 (sense: 5′-AAC TAT TGC TTC AGC TCC ACA GAG-3′, anti-sense: 5′-AGT TGG ATG GTA GCC CTT G-3′, 210 bp), α-SMA (sense: 5′-GTG ACT ACT GCC GAG CGT G-3′, anti-sense: 5′-ATA GGT GGT TTC GTG GAT GC-3′, 250 bp), PDGFR-β (sense: 5′-CAT CAT GAG GGA CTC AAA CT-3′, anti-sense: 5′-GAT GGC ATT GTA GAA CTG GT-3′, 230 bp), VEGF (sense: 5′-CAC TGG ACC CTG GCT TTA CT-3′, anti-sense: 5′-GGT GAT GTT GCT CTC TGA CG-3′, 290 bp), and β-actin (sense: 5′-TGT CCA CCT TCC AGC AGA TGT-3′, anti-sense: 5′-TGT CCC TGT ATG CCT CTG GT-3′, 101 bp). For ACTA2, COL1A1, and LOXL2, TaqMan Gene Expression Assays (Applied Biosystems) were performed according to the manufacturer’s instructions.

### 2.8. Collagen and Fibrosis Assays

Collagen contraction assays were performed using the CytoSelect 24-Well Cell Contraction Assay Kit (Cell Biolabs, Inc., San Diego, CA, USA) according to the manufacturer’s instructions. MRC5, LL24, and LL97A fibroblasts were treated with TGF-β1 (5 ng/mL) in the presence or absence of Compound **#765** (100 nM), and gel contraction was monitored at 24, 48, and 72 h (*n* = 3). Contraction was quantified using ImageJ software (NIH). Soluble collagen content was measured in MRC5 fibroblasts using the Sircol collagen assay kit (Biocolor, Belfast, UK), following the manufacturer’s protocol. Cell viability was assessed using the CCK-8 assay (Dojindo, Tokyo, Japan), and no significant cytotoxicity was observed under the treatment conditions used (Supplementary Figure S4).

### 2.9. Animal Disease Model and Histological Analysis

Twelve-week-old male C57BL/6 mice were purchased from Orient Bio (Seoul, Republic of Korea) and maintained in controlled animal care facilities under specific pathogen-free conditions, with sterilized food and water provided ad libitum. All procedures were approved by the Institutional Animal Care and Use Committee of Yonsei University College of Medicine (approval no. IACUC# 2019-0101). Clara cell 10-kD protein (CC10)-tTS-rtTA-TGF-β1 transgenic mice were also used (IACUC# 2020-0108). The CC10 promoter specifically targets bioactive TGF-β1 expression in the lungs [26,27]. Transgenic mice were confirmed by PCR genotyping, as described previously [28]. TGF-β1 overexpression was induced by administering doxycycline (Dox; Cat#D9891, Sigma-Aldrich) in drinking water (0.5 mg/mL) supplemented with 5% sucrose. Dox-containing water was provided ad libitum throughout the experimental period. The indicated concentrations of Compound **#765** were administered daily by oral gavage, with both Dox water and Compound **#765** supplied for one month. For the bleomycin (BLM)-induced pulmonary fibrosis model, mice were divided into five groups: negative control, BLM only, and three groups treated with Compound **#765** (1, 10, and 30 mg/kg). Compound **#765** was administered orally five days/week, beginning one day before the BLM injection. Mice were anesthetized with Zoletil 50 (0.015 mL/20 g; Virbac, Carros, France) and Rompun (0.5 mg/20 g; Bayer Korea, Seoul, Republic of Korea), and pulmonary fibrosis was induced by a single intratracheal instillation of BLM (1.5 U/kg in 50 μL of sterile saline). The negative control group received only saline. Compound **#765** was administered for 2 or 3 weeks until the mice were euthanized.

Post-Euthanasia Procedures and Histological Analysis: Following euthanasia, bronchoalveolar lavage fluid (BALF) was collected by cannulating the trachea with a polyethylene catheter (BD Biosciences, San Jose, CA, USA) and instilling 1 mL sterile PBS once. The recovered BALF was centrifuged at 300× *g* for 5 min at 4 °C, and total cell numbers were determined using a hemocytometer after Trypan Blue staining (Gibco, Waltham, MA, USA). Subsequently, pulmonary circulation was perfused with 10 mL sterile PBS via the right ventricle, after which the lungs were rinsed and fixed overnight in 4% formaldehyde at 4 °C. Tissues were processed using graded ethanol and xylene and embedded in paraffin. Sections (5 μm) were stained with hematoxylin and eosin (H&E) and Masson’s trichrome (MT) according to the manufacturer’s protocols. For immunohistochemistry, antigen retrieval was performed using the standard procedure. The sections were incubated with anti-LOXL2 (1:100; Santa Cruz Biotechnology, Santa Cruz, CA, USA), anti-α-SMA (1:500; Santa Cruz Biotechnology), or anti-collagen I (1:1000; Abcam, Cambridge, UK) antibodies, followed by HRP-conjugated secondary antibodies. Soluble collagen in the lung tissues was quantified using a Sircol collagen assay kit (Biocolor, Belfast, UK). For second-harmonic-generation (SHG) imaging, the sections were baked at 60 °C for 1 h, deparaffinized, cleared in xylene, passed through a graded ethanol series for dehydration, and finally mounted with Permount (Fisher Chemical, Pittsburgh, PA, USA) without additional staining. Imaging was performed using an LSM7MP multiphoton microscope (Carl Zeiss, Oberkochen, Germany) with 780 nm excitation and a 430–450 nm detection window. All animal experiments were performed using biologically independent samples (*n* = 3–5 per group) to ensure statistical robustness and reproducibility.

### 2.10. Statistical Analysis

Data are expressed as mean ± standard deviation (SD). Statistical analyses were performed using GraphPad Prism software (versions 5–9; GraphPad Software, San Diego, CA, USA). One-way analysis of variance (ANOVA) or unpaired Student’s *t*-tests were applied as appropriate, with *p* < 0.05 considered statistically significant. All experiments were performed in at least three independent biological replicates.

## 3. Results

### 3.1. Synthesis, Characterization, and Activity of Compound #765

We synthesized a novel indolizine derivative, Compound **#765** (6-Amino-5-(4-methoxybenzoyl)indolizine-7-carbonitrile, Figure 1A), which was identified through the extensive screening of numerous candidate compounds based on LOXL2 inhibitory activity and was subsequently confirmed to inhibit LOXL2 enzymatic activity. The chemical identity of Compound **#765** was confirmed by spectroscopic analysis. The ^1^H and ^13^C{1H} NMR spectra (Supplementary Figure S1) were consistent with the expected structure, and the high-resolution mass spectrometry (HRMS) data further supported the molecular formula, yielding *m*/*z* [M + H] + calcd. for C_17_H_14_N_3_O_2_ 292.1081, found 292.1085 (Supplementary Figure S2). The purity of Compound **#765** was determined to be 99.42% in the HPLC analysis (λ = 254 nm) (Supplementary Figure S3), collectively confirming the chemical structure of Compound **#765** and its high purity for subsequent biological studies.

The DARTS assay provided evidence consistent with an interaction between Compound **#765** and LOXL2, as indicated by its protection from protease-induced degradation (Figure 1B). Consistent with this interaction, Compound **#765** selectively inhibited the deaminase activity of LOXL2, while showing no effect on LOX activity under identical assay conditions (Figure 1C). To exclude the possibility that Compound **#765** functions as an antioxidant, we measured hydrogen peroxide levels in the absence of LOXL2 and observed no changes (Figure 1D). In silico docking analysis using SwissDock and PolMol showed that Compound **#765** preferentially binds to the catalytic groove of LOXL2 (ΔG ≈ −7.0 kcal/mol). This theoretically predicted affinity was greater than that predicted for other LOX family members (Supplementary Table S1), supporting the observed selectivity. Collectively, these results support that Compound **#765** interacts with LOXL2 and inhibits its enzymatic activity, consistent with reducing H_2_O_2_ generation.

### 3.2. Compound #***765*** Treatment Inhibits Collagen Synthesis and Contraction in Lung Fibroblasts

**Figure 2:**
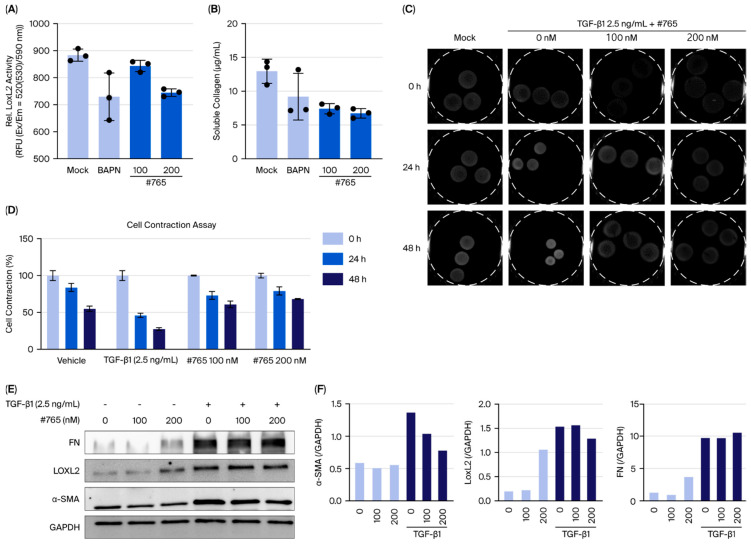
Compound **#765** inhibits LOXL2 activity and TGF-β1-induced collagen remodeling in lung fibroblasts. (**A**,**B**) Effects of Compound **#765** in the absence of TGF-β1. (**A**) LOXL2 enzymatic activity in MRC5 fibroblast supernatants after 72 h of treatment with Compound **#765** or BAPN, showing a dose-dependent decrease. (**B**) Soluble collagen levels were significantly reduced following Compound **#765** treatment. (**C**–**F**) Effects of Compound **#765** under TGF-β1 stimulation (2.5 ng/mL). (**C**) Representative images of collagen gel contraction in MRC5 fibroblasts with TGF-β1 in the presence or absence of Compound **#765** (100 nM or 200 nM). Gel contraction assays were performed in 24-well plates. The gels were then carefully detached, transferred to 60 mm dishes, and images were acquired using a Fusion Solo 6S system equipped with an eVo-6 camera. (**D**) Quantification of gel contraction at 0, 24, 48 h using ImageJ. (**E**) Immunoblot analysis of fibrosis-related proteins (α-SMA, LOXL2, and fibronectin (FN)) in TGF-β1-treated MRC5 cells with or without Compound **#765**. (**F**) Densitometric quantification of protein expression normalized to GAPDH. Overall, Compound **#765** inhibited LOXL2 enzymatic activity, reduced collagen secretion, suppressed TGF-β1-induced collagen contraction, and downregulated α-SMA and LOXL2 while FN levels remained largely unchanged. Data are presented as mean ± SD (*n* = 3). LOXL2, lysyl oxidase-like 2; TGF-β1, transforming growth factor-beta 1; MRC5, human lung fibroblast cell line; α-SMA, alpha-smooth muscle actin; FN, fibronectin.

We examined the effect of Compound **#765** on LOXL2 enzymatic activity and soluble collagen production in fibroblasts. After 72 h of treatment, LOXL2 activity in the human fetal lung fibroblast (MRC5) cell culture supernatant showed a clear decrease (Figure 2A). Collagen secretion into the culture supernatant was markedly reduced in Compound **#765**-treated cells (Figure 2B). Additionally, we assessed collagen contraction in MRC5 cells treated with Compound **#765** in the presence of 2.5 ng/mL TGF-β1. Compound **#765** effectively suppressed TGF-β1-induced collagen contraction in a dose-dependent manner at 100 nM and 200 nM, with effects lasting up to 48 h (Figure 2C,D). Furthermore, Compound **#765** downregulated fibrosis-related proteins, including α-SMA and LOXL2, but exhibited no significant effect on fibronectin (FN) levels (Figure 2E,F). Importantly, no significant cytotoxicity was observed at the concentrations used in these experiments, confirming that the observed antifibrotic effects were not due to reduced cell viability (Supplementary Figure S4). These findings indicate that Compound **#765** inhibits LOXL2 enzymatic activity, reduces collagen secretion, and suppresses TGF-β1-induced collagen contraction in vitro.

### 3.3. Compound #***765*** Exhibits Potent Anti-Fibrotic Activity in Multiple Mouse Models of Pulmonary Fibrosis

**Figure 3:**
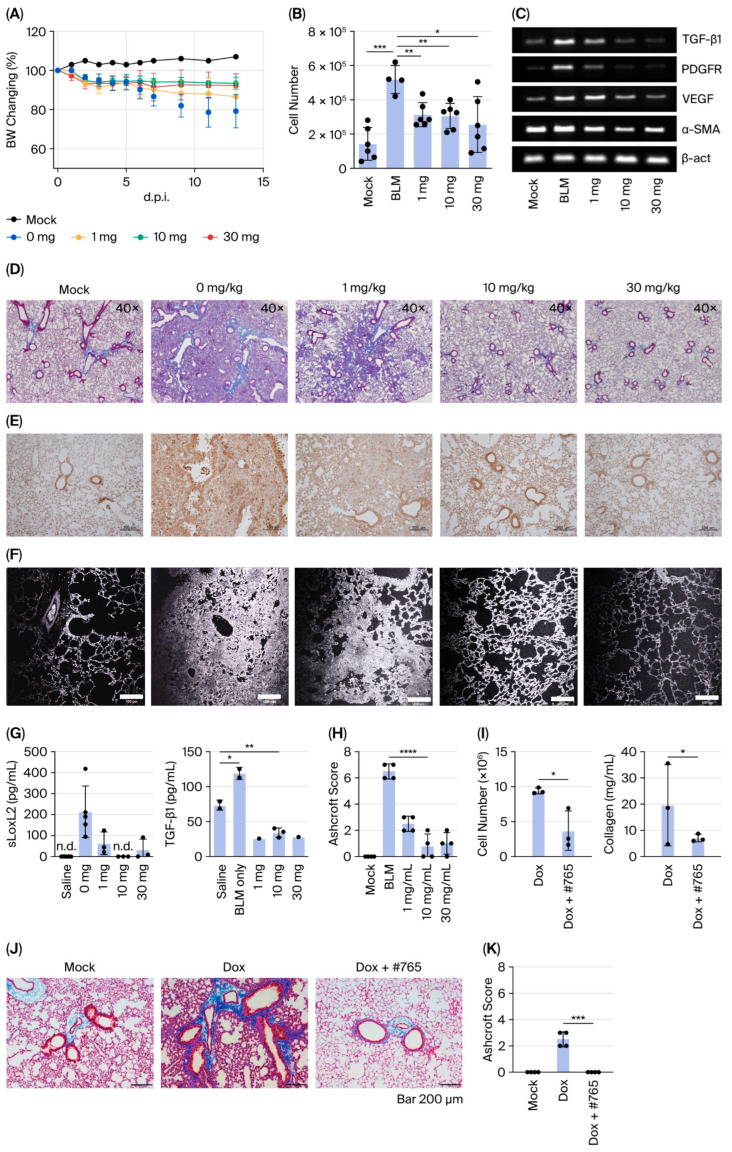
Compound **#765** attenuates pulmonary fibrosis in bleomycin- and TGF-β1 transgenic mouse models. (**A**–**G**) Bleomycin (BLM)-induced pulmonary fibrosis model. (**A**) Body weight changes in C57BL/6 mice for 14 days after intratracheal (i.t.) administration of BLM (1.5 U/kg), with or without Compound **#765** (1, 10, or 30 mg/kg; oral, 5 days/week). (**B**) Total cell numbers in bronchoalveolar lavage fluid (BALF) at day 14. (**C**) mRNA expression of fibrosis-related genes (TGF-β1, PDGFR, VEGF, and α-SMA) in lung tissues. (**D**) Representative Masson’s trichrome (MT) staining showing collagen deposition (blue). Groups: mock (saline only), 0 mg/kg (BLM only), and BLM + Compound **#765** (1, 10, or 30 mg/kg). Scale bar = 40×. (**E**) Second harmonic generation (SHG) imaging of fibrillar collagen. Scale bar = 100 μm. (**F**) Immunohistochemistry of LOXL2 expression (brown). Scale bar = 100 μm. (**G**) Quantification of LOXL2 in BALF and TGF-β1 protein levels in lung tissue by ELISA. Data = mean ± SD (*n* = 3–5). * *p* < 0.05, ** *p* < 0.01 vs. BLM only. (**H**) Ashcroft scores of MT-stained lung sections from (**D**) in the BLM model. **** *p* < 0.0001 vs. BLM only. (**I**–**K**) Chronic TGF-β1 transgenic mouse model. Lung-specific TGF-β1 overexpression was induced by doxycycline (Dox). Mice were treated with Compound **#765** (30 mg/kg, 4 weeks) or vehicle. (**I**) Quantification of BALF total cell counts and soluble collagen in lung homogenates from Dox-induced transgenic mice (*n* = 3). * *p* < 0.05 vs. Dox only. (**J**) Representative MT-stained lung sections. Mock: no Dox; Dox: TGF-β1-induced pulmonary fibrosis; Dox + **#765**: Compound **#765** treatment following TGF-β1 induction. Scale bar = 200 μm. (**K**) Ashcroft scores of MT-stained lung sections from (**J**) in the BLM model. *** *p* < 0.001 vs. Dox only. BLM, bleomycin; BALF, bronchoalveolar lavage fluid; TGF-β1, transforming growth factor-beta 1; PDGFR, platelet-derived growth factor receptor; VEGF, vascular endothelial growth factor; α-SMA, alpha-smooth muscle actin; ELISA, enzyme-linked immunosorbent assay; SD, standard deviation; n.d., not detected.

To evaluate the in vivo efficacy of Compound **#765** in inhibiting LOXL2 enzymatic activity and fibrotic protein production, pulmonary fibrosis was induced in C57BL/6 mice via intratracheal administration of 1.5 U/kg bleomycin (BLM). Mice were orally administered Compound **#765** (1, 10, or 30 mg/kg, 5 days per week) starting one day before BLM injection and monitored for 14 days. Notably, Compound **#765** has a relatively short half-life in vivo, with 1.7 h following oral administration and 0.6 h following intravenous administration (Supplementary Table S2), indicating rapid systemic clearance. Based on these pharmacokinetic characteristics, Compound **#765** was administered daily. Despite its short half-life, daily administration resulted in significant antifibrotic efficacy in both pulmonary fibrosis models. Compound **#765** significantly alleviated BLM-induced body weight loss and inflammatory cell infiltration in bronchoalveolar lavage fluid (BALF) (Figure 3A,B). Expression of fibrosis-related genes (TGF-β1, PDGFR, VEGF, and α-SMA) was dose-dependently downregulated (Figure 3C). Histological analysis using Masson’s trichrome staining revealed marked improvement, with reduced inflammatory foci and collagen deposition (Figure 3D,H). Second-harmonic-generation (SHG) imaging further confirmed the reduced deposition of cross-linked collagen fibers, consistent with MT staining (Figure 3E). At 30 mg/kg, lung morphology was nearly indistinguishable from that of the control mice. Immunohistochemistry showed that Compound **#765** decreased the number of LOXL2-positive immune cells in a dose-dependent manner (Figure 3F), accompanied by lower soluble LOXL2 and TGF-β1 protein levels in BALF (Figure 3G). To validate these findings in a genetic model, doxycycline-induced TGF-β1 transgenic mice were treated with Compound **#765**, which markedly attenuated inflammatory cell infiltration, fibrotic foci, and extracellular matrix deposition (Figure 3I–K) [29]. Together, these results demonstrate that Compound **#765** exerts potent, broad-spectrum anti-fibrotic effects in both BLM-induced and TGF-β1-driven pulmonary fibrosis models.

### 3.4. Compound #***765*** Treatment Inhibits TGF-β1-Induced Activation of Lung Fibroblasts Derived from Patients with IPF

**Figure 4:**
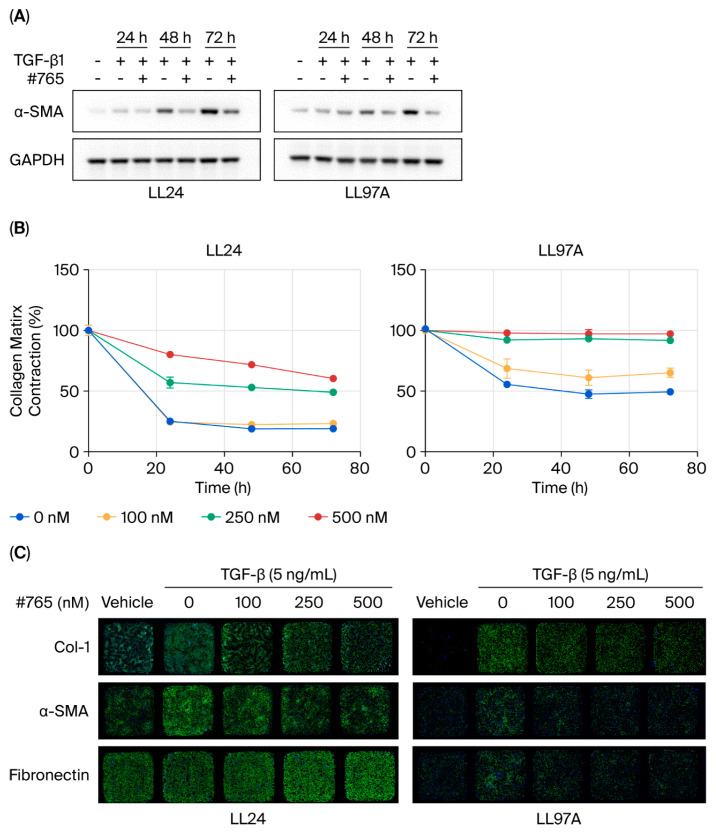
Compound **#765** inhibits fibrosis-related protein expression and collagen contraction in human lung fibroblasts. Normal lung fibroblasts (LL24) and IPF-derived fibroblasts (LL97A) were stimulated with TGF-β1 (5 ng/mL) and treated with Compound **#765**. (**A**) Representative Western blot showing α-SMA expression after treatment with Compound **#765** (100 nM) for 24, 48, and 72 h. GAPDH served as a loading control. (**B**) Collagen gel contraction assay in LL24 and LL97A fibroblasts treated with Compound **#765** (100, 250, and 500 nM) following TGF-β1 stimulation. Contraction was quantified at the indicated time points. Data are presented as mean ± SD (*n* = 3). (**C**) Representative immunofluorescence images of collagen I (Col-I), α-SMA, and FN after 72 h of treatment with TGF-β1 (5 ng/mL) and Compound **#765** (100, 250, and 500 nM). Nuclei were counterstained with Hoechst 33342 (blue). Images were acquired at 40× magnification. α-SMA, alpha-smooth muscle actin; LL24, a normal lung fibroblast cell line; IPF, idiopathic pulmonary fibrosis; LL97A, an IPF-derived fibroblast cell line; TGF-β1, transforming growth factor-beta 1; Col-I, collagen I; FN, fibronectin.

**Figure 5:**
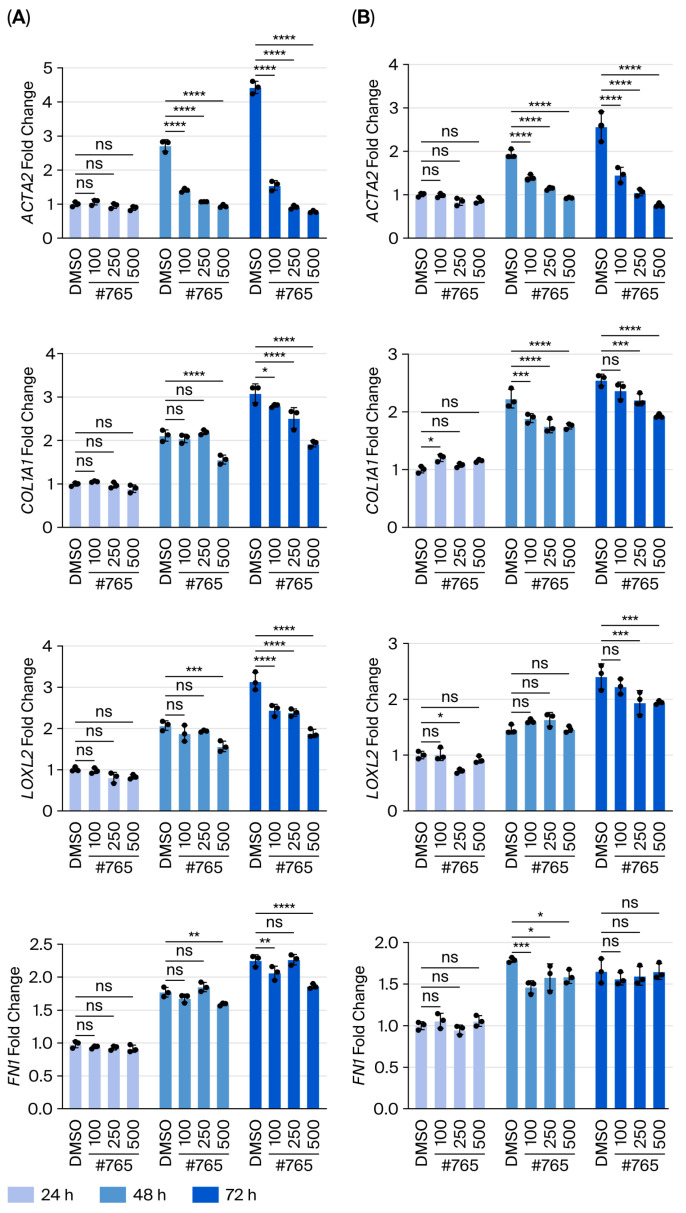
Compound **#765** suppresses TGF-β1-induced fibrotic gene expression in human lung fibroblasts. Normal lung fibroblasts (LL24) and IPF-derived fibroblasts (LL97A) were stimulated with TGF-β1 (5 ng/mL) and treated with Compound **#765** (100, 250, and 500 nM) for 24, 48, and 72 h. mRNA expression of fibrosis-associated genes was measured by quantitative real-time PCR. (**A**) Relative mRNA levels of ACTA2, COL1A1, and LOXL2 in LL24 cells. (**B**) Relative mRNA levels of ACTA2, COL1A1, and LOXL2 in LL97A cells. Compound **#765** significantly reduced TGF-β1-induced gene expression in dose- and time-dependent manner in both cells. Data are presented as mean ± SD (*n* = 3–5). * *p* ≤ 0.05, ** *p* ≤ 0.01, *** *p* ≤ 0.001, and **** *p* ≤ 0.0001, vs. TGF-β1 + DMSO group. ns, not significant. ACTA2, actin alpha 2, smooth muscle; COL1A1, collagen type I alpha 1.

To investigate the effects of Compound **#765**, we analyzed its impact on normal lung fibroblasts (LL24) and IPF-derived fibroblasts (LL97A). Cells were treated with 2.5 ng/mL TGF-β1, a concentration optimized for these cell lines, in the presence or absence of 100 nM Compound **#765**. Treatment with Compound **#765** markedly reduced α-SMA protein expression at 24, 48, and 72 h in both cell types (Figure 4A). This downregulation was consistently observed across all treated groups and confirmed by immunofluorescence (IF) staining (Figure 4C, and Supplementary Figure S5). Immunofluorescence analysis further demonstrated the differential regulation of collagen I and FN depending on the fibroblast origin. In LL97A (IPF-derived) fibroblasts, Compound **#765** markedly decreased collagen I and α-SMA levels, with FN protein deposition reduced despite relatively unchanged mRNA levels (Figure 4C and Figure 5B). In contrast, LL24 cells exhibited only modest reductions in collagen I and FN, suggesting a more selective anti-fibrotic effect in diseased fibroblasts (Figure 4C and Figure 5A). Fibronectin levels showed differential regulation across fibroblast types. In LL24 cells, FN mRNA levels decreased, whereas immunofluorescence staining indicated minimal change at the protein level. In contrast, in IPF-derived fibroblasts (LL97A), immunofluorescence staining demonstrated a reduction in FN protein levels, while FN mRNA levels remained largely unchanged. These findings suggest that FN regulation may be influenced by post-transcriptional or extracellular matrix-associated mechanisms beyond direct LOXL2 inhibition. A collagen contraction assay further supported these findings, showing that even low doses of Compound **#765** inhibited TGF-β1-induced ECM contraction in both LL24 and LL97A cells, with a tendency toward greater inhibition in IPF-derived LL97A fibroblasts (Figure 4B). These results confirmed that Compound **#765** consistently inhibits collagen contraction in both cell lines. These observations indicate that Compound **#765** preferentially attenuates fibrotic remodeling in patient-derived cells.

Consistent with these results, quantitative gene-expression analysis revealed dose-dependent reductions in ACTA2, COL1A1, and LOXL2 mRNA levels in both LL24 (Figure 5A) and LL97A (Figure 5B) fibroblasts. However, FN mRNA levels remained largely unchanged in LL97A fibroblasts despite the reduction observed by IF staining, suggesting that FN regulation may involve additional post-transcriptional mechanisms. Collectively, these findings demonstrate that Compound **#765** exerts potent antifibrotic effects in lung fibroblasts, with particularly pronounced efficacy in IPF-derived cells, underscoring its translational potential.

## 4. Discussion

Pulmonary fibrosis, including IPF, is a chronic, progressive lung disease characterized by excessive scarring and pathological remodeling of the lung tissue. Despite the ATS/ERS/JRS/ALAT guidelines recommending various drug combinations, available therapeutics remain limited [11]. Existing antifibrotic agents, such as pirfenidone and nintedanib, primarily delay disease progression and have restricted efficacy. Nintedanib restores microvascular architecture and mitigate inflammation and fibrosis-related features in murine models [30]. Pirfenidone also exerts anti-inflammatory and antioxidant effects, although it is associated with adverse events, including dyspepsia, anorexia, and weight loss, which frequently lead to dose reduction or the discontinuation of treatment [7]. These limitations emphasize the urgent need for novel antifibrotic agents capable of both attenuating and reversing fibrotic remodeling with improved safety and pharmacokinetic properties. Small-molecule inhibitors like Compound **#765** offer this potential due to enhanced tissue penetration, favorable bioavailability, and reduced immunogenicity compared with biologics.

LOXL2 facilitates collagen cross-linking, particularly within the 7S region of Type IV collagen, forming the structural framework of basement membranes [31]. LOXL2 is overexpressed in fibrotic lesions and various cancers, where it promotes epithelial–mesenchymal transition (EMT) and matrix remodeling [32,33,34]. LOXL2 enhances EMT through both Snail1-dependent and independent mechanisms while downregulating tight junction molecules, thereby contributing to tissue remodeling and fibrotic progression [35,36]. Furthermore, TGF-β plays a pivotal role in EMT, and LOXL2 expression increases in a time- and dose-dependent manner in response to TGF-β stimulation [37]. While EMT in cancer is often reversible, fibrosis is irreversible, underscoring the need for distinct therapeutic approaches. Previous clinical attempts to target LOXL2 using antibody-based therapies, such as simtuzumab, have been unsuccessful, mainly due to poor tissue penetration or compensatory fibrotic pathways [20,38,39]. Several LOX/LOXL2-targeted inhibitors have been investigated in pulmonary fibrosis. Early efforts included BAPN, a non-selective LOX inhibitor, which showed antifibrotic effects but was limited by reported adverse effects (e.g., skeletal abnormalities and metabolic changes) and a lack of specificity [40]. Recent preclinical studies of small molecules have shown mixed success, primarily due to short half-lives or incomplete selectivity [41]. In this study, we synthesized a series of indolizine derivatives and evaluated their LOXL2 inhibitory activity. Through this rigorous process, Compound **#765** was identified as a lead candidate with potent inhibitory activity. Compared to antibody-based approaches, small-molecule compounds such as **#765** may offer potential advantages, including improved tissue penetration and favorable pharmacokinetic properties. In addition, compound **#765** demonstrated effective interaction with LOXL2 in our experimental systems [21]. We found that Compound **#765** interacts with LOXL2 and inhibits its enzymatic activity (Figure 1). While Compound **#765** may interact with other LOX isoforms (Supplementary Table S1), this study focuses primarily on its interaction with LOXL2, the principal enzymatic target identified. This LOXL2 inhibition likely contributes to the mechanistic basis for the observed antifibrotic effects.

Preliminary safety and pharmacokinetic assessments (including LC/MS observation) suggested that Compound **#765** was well tolerated and rapidly cleared in vivo. Despite its relatively short half-life (1.7 h for oral and 0.6 h for intravenous administration), it exhibited significant therapeutic efficacy in both BLM-induced and TGF-β1-driven pulmonary fibrosis models (Figure 3). Importantly, even when Compound **#765** was administered only during the inflammatory phase or during the established fibrotic phase, it still effectively delayed fibrosis progression, suggesting that its antifibrotic action is not restricted to early prophylactic intervention (Supplementary Figure S5). This result suggests that Compound **#765** may overcome some of the limitations associated with earlier LOX-targeted agents and that its anti-fibrotic effects are not solely dependent on prolonged systemic circulation, a common limitation for many biologic therapies. Compound **#765** also suppressed fibrosis-associated markers in fibroblasts derived from IPF patients (Figure 4 and Figure 5), highlighting its translational potential and suggesting a selective therapeutic benefit in diseased cells. Mechanistically, Compound **#765** exerts its antifibrotic effects, at least in part, through the inhibition of LOXL2 enzymatic activity, with secondary modulation of TGF-β-associated fibrotic signaling. This effect can be understood in the context of extracellular matrix (ECM) remodeling. LOXL2 catalyzes collagen crosslinking, contributing to ECM stiffening, which promotes fibroblast activation and sustains profibrotic signaling. By inhibiting LOXL2, Compound **#765** likely reduces ECM crosslinking and stiffness, thereby attenuating fibroblast activation and disrupting the positive feedback loop that drives fibrotic progression. Given the central role of TGF-β in fibroblast activation, ECM deposition, and collagen turnover, the inhibition of LOXL2 and the associated attenuation of TGF-β responses further support the therapeutic potential of compound **#765** in pulmonary fibrosis and IPF. Our data suggest that LOXL2 targeting is an important mechanism, with decreases in TGF-β-related markers likely occurring secondarily to ECM remodeling rather than the direct suppression of canonical TGF-β/Smad signaling [12,42]. Importantly, inhibiting enzymatic activity does not necessarily require changes in expression levels, which may explain the limited changes observed in LOXL2 expression in vivo. Further studies of downstream phosphorylation events (e.g., Smad2/3, MAPK) will be necessary, but current preclinical data support the rationale to continue developing Compound **#765**.

Comprehensive toxicological and pharmacokinetic studies are therefore required to fully define its safety profile and therapeutic potential. The confirmation of its therapeutic potential and clinical applicability mandates further efficacy studies using large animal models and human systems. Despite this limitation, Compound **#765** shows promise in preventing fibrosis development and has broader potential in treating established disease.

In summary, this study demonstrates that Compound **#765** exerts antifibrotic effects by inhibiting LOXL2 enzymatic activity and its downstream impact on fibrotic remodeling as follows:

(i) Identification as a potent LOXL2 enzymatic inhibitor;
(ii) Reduction in lung fibrosis progression both in vitro and in vivo through decreased collagen production and ECM remodeling;
(iii) Alleviating pulmonary fibrosis features, including reduced collagen accumulation and inflammatory cell infiltration;
(iv) Consistent antifibrotic efficacy across different murine models, including bleomycin-induced acute fibrosis and TGF-β-driven chronic fibrosis.

## Data Availability

The datasets used and/or analyzed in the current study are available from the corresponding author upon reasonable request. However, due to ethical restrictions (regulation by Institutional Review Board), they are not publicly available.

## Supplementary Materials

The following supporting information can be downloaded at https://www.mdpi.com/article/10.3390/pharmaceutics18050554/s1. Table S1. Comparative docking and experimental validation summary for compound **#765** against the LOX family. Table S2. Pharmacokinetic Parameters of Compound **#765**. Figure S1. ^1^H and ^13^C NMR spectra of compound **#765**. Figure S2. HRMS spectrum of compound **#765**. Figure S3. HPLC chromatogram of Compound **#765**. The purity of compound **#765** was determined to be 99.4% by HPLC analysis (λ = 254 nm). Figure S4. Cytotoxicity test of Compound **#765** in lung fibroblast. Figure S5. Histological evaluation of preventive antifibrotic effects of Compound **#765** in the bleomycin-induced pulmonary fibrosis model.
