# Supplementary material for: Targeting Lysyl Oxidase-like 2: A Therapeutic Strategy for Idiopathic Pulmonary Fibrosis with a Novel Indolizine Derivative
Source: Pharmaceutics. 2026 Apr 30;18(5):554. doi: 10.3390/pharmaceutics18050554 (PMC13211120; doi:10.3390/pharmaceutics18050554)
Supplement: Supplementary file 1 [file pharmaceutics-18-00554-s001.zip › pharmaceutics-4189881-supplementary.pdf]

Supplementary Data

Materials and Methods

Computational Methods for Protein-Ligand Docking and Analysis of Compound #765 Binding to LOXL2

The 3D structures of human lysyl oxidase (LOX) and its homologs (LOXL1–4) were obtained from the Protein Data Bank (PDB) and AlphaFold Protein Structure Database, with predicted models used for LOXL1–4 due to the lack of experimentally resolved structures. Compound #765, an indolizine derivative, was drawn using ChemDraw, converted to 3D format with OpenBabel, and optimized using SwissParam for force field parameters. Molecular docking was performed with SwissDock using the EADock DSS algorithm in blind docking mode to explore potential binding sites, with binding free energies (ΔG) reported as SwissParam Scores. Docking poses were visualized with PolMol, and interactions within 5 Å of the ligand were analyzed manually for hydrogen bonds and hydrophobic interactions. Computational docking was executed using PyMOL and SwissDock web interfaces. AI-assisted guidance was used only for general procedural steps, such as selecting appropriate docking tools and organizing the workflow. No AI tools were involved in docking calculations, data analysis, or scientific interpretation.

Pharmacokinetics of Compound #765

The pharmacokinetics of Compound #765 were evaluated in ICR mice, revealing that the compound exhibits a relatively short half-life *in vivo*. Following oral (PO) administration, the half-life of Compound #765 was found to be 1.7 hours, while intravenous (IV) administration resulted in a half-life of 0.6 hours. Despite the rapid clearance of Compound #765 from the body, the compound demonstrated significant efficacy in both BLM-induced and TGF-β1-driven pulmonary fibrosis models, suggesting that the drug's therapeutic effects are not solely dependent on prolonged systemic circulation. The data were obtained through collaboration with the Korea Research Institute of Bioscience and Biotechnology (KRIBB).

Supplementary Table S1. Comparative docking and experimental validation summary for compound #765 against the LOX family.

Protein	ΔG (SwissDock, kcal mol⁻¹)	Docking pose (PolMol analysis)	Key interacting residues (PolMol)	Experimental validation (DARTS)	Notes / structural state
**LOX**	–6.0	Ligand located on the protein surface without deep pocket insertion	None observed	Not detected	Catalytic pocket not engaged; likely non-specific binding
**LOXL1**	–7.65	Ligand buried in a hydrophobic cavity near the catalytic site	Phe458, Tyr461, Asp462, Arg425	Not tested	Strongest predicted binder among the LOX family; catalytic domain contact
**LOXL2**	**–7.0 (range –6.9 to –7.0)**	**Ligand stably accommodated in the catalytic groove**	**Glu375, Arg425, Tyr461**	**Protease protection confirmed**	**Direct binding validated by DARTS; likely specific to mature form**
**LOXL3**	- 6.93	Ligand embedded within a shallow groove adjacent to the active site	His540, Glu550	Not tested	Moderate affinity; possible allosteric interaction pattern
**LOXL4**	–6.6	Ligand located near the catalytic region forming hydrophobic contacts	Phe455, Arg460, Tyr465	Not tested	Moderate binding adjacent to the catalytic pocket

ΔG values were obtained using the SwissDock web server, and docking poses were visualized using the PolMol platform.

DARTS results confirmed direct binding to LOXL2, supporting the *in silico* prediction of preferential interaction with the catalytically active form of LOXL2.

Supplementary Table S2. Pharmacokinetic Parameters of Compound #765.

PK Parameter		Compound #765 (IV route)			Compound #765 (PO route)		
		Mean	SD	N	Mean	SD	N
Dose	(mg/kg)	2		3	10		3
t_{max}	(hr)	NA			0.4	0.1	3
C_{max}	(ng/ml)	NA			161.3	45.5	3
AUC_{last}	(ng.hr/ml)	325.67	95.80	3	231.7	45.2	3
AUC_{int}	(ng.hr/ml)	357.14	110.47	3	288.5	75.1	2
CL	(L/hr/kg)	6.00	1.95	3	NA		
V_{ss}	(L/kg)	2.9	1.10	3	NA		
V_z	(L/kg)	4.83	1.87	3	NA		
$t_{1/2}$	(hr)	0.61	0.30	3	1.7	0.0	2
MRT_{inf}	(hr)	0.53	0.26	3	2.2	0.0	2
F	(%)	NA			16.2	4.2	

This table summarizes the pharmacokinetic (PK) parameters of Compound #765 following administration via intravenous (IV) and intra-peritoneal (IP) routes. The data include the mean, standard deviation (SD), and number of subjects (N) for each parameter. The parameters are as follows:

Dose: The amount of Compound #765 administered per kilogram of body weight.

t_{max}: The time (in hours) at which the maximum plasma concentration (C_{max}) occurs.

C_{max}: The maximum concentration of Compound #765 in the plasma (ng/ml).

AUC_{last}: The area under the plasma concentration-time curve from time 0 to the last measurable concentration (ng·hr/ml).

AUC_{inf}: The area under the plasma concentration-time curve from time 0 to infinity (ng·hr/ml).

CL: The clearance rate of Compound #765 from the body (L/hr/kg).

V_{ss}: The steady-state volume of distribution (L/kg).

V_z: The terminal volume of distribution (L/kg).

$t_{1/2}$: The half-life of Compound #765 (hr), the time it takes for half of the drug to be eliminated from the body.

MRT: The mean residence time of the drug in the body (hr).

F: The bioavailability of Compound #765, expressed as a percentage.

NA: not available

Supplementary Figure S1. ^{1}H and ^{13}C NMR spectra of compound #765.

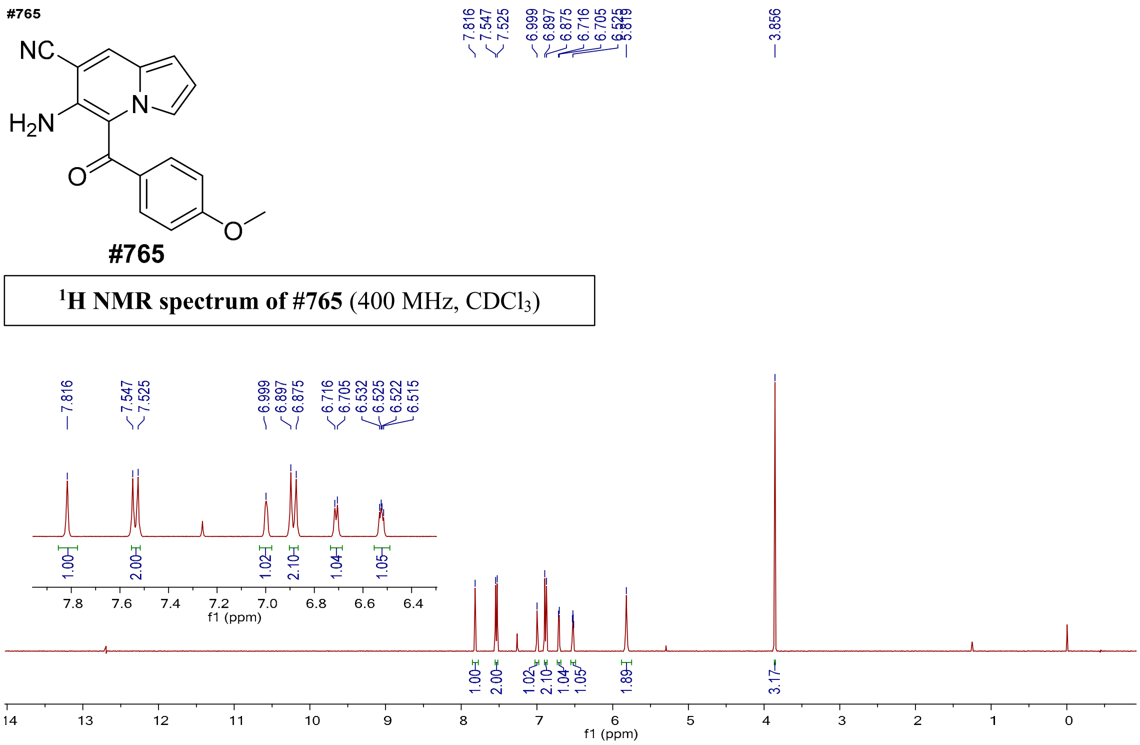

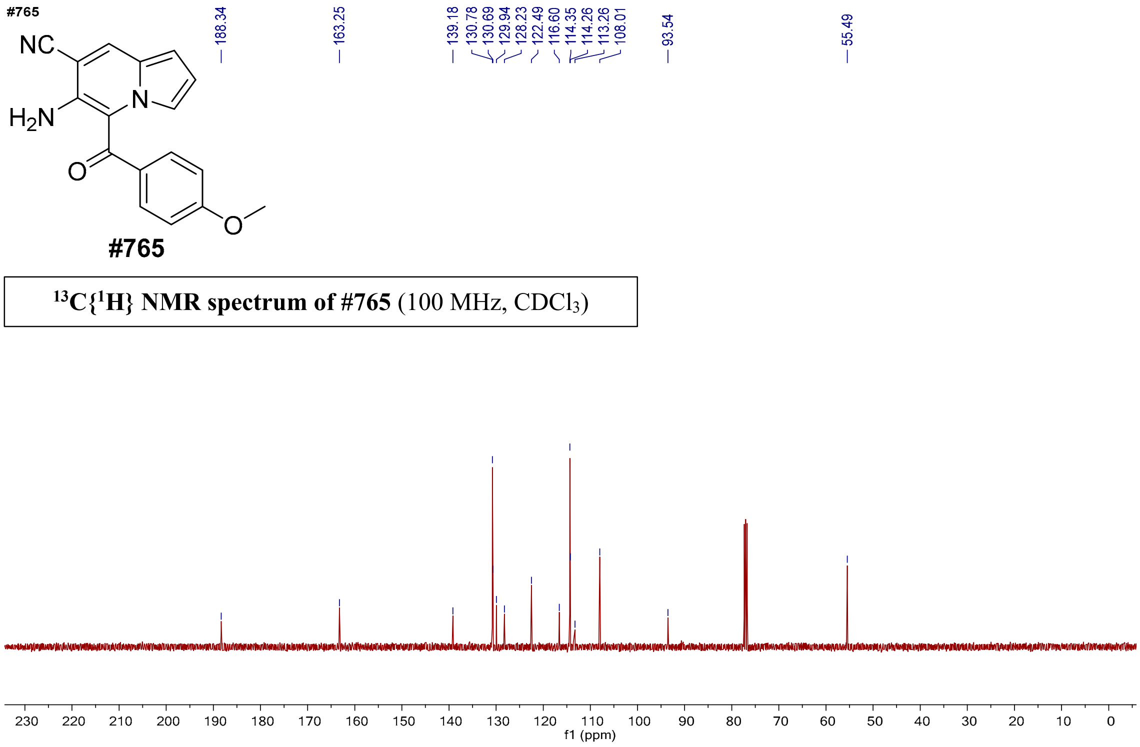

Supplementary Figure S2. HRMS spectrum of compound #765

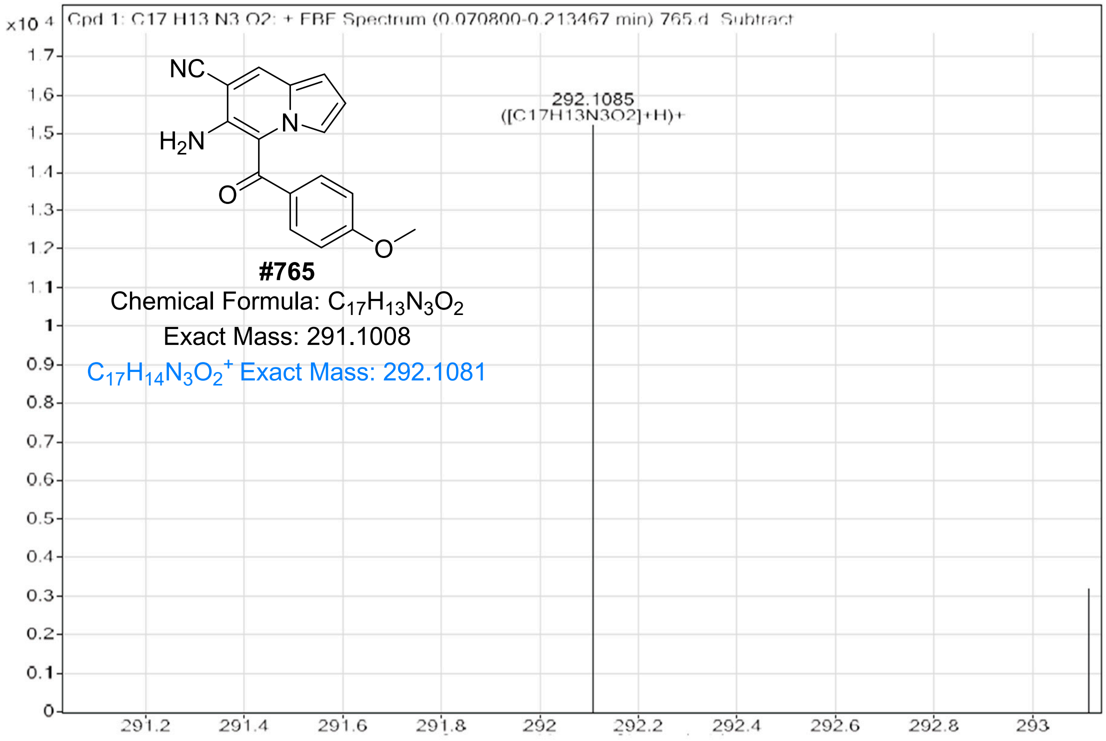

Supplementary Figure S3. HPLC chromatogram of Compound #765. The purity of compound **#765** was determined to be 99.4% by HPLC analysis (λ = 254 nm).

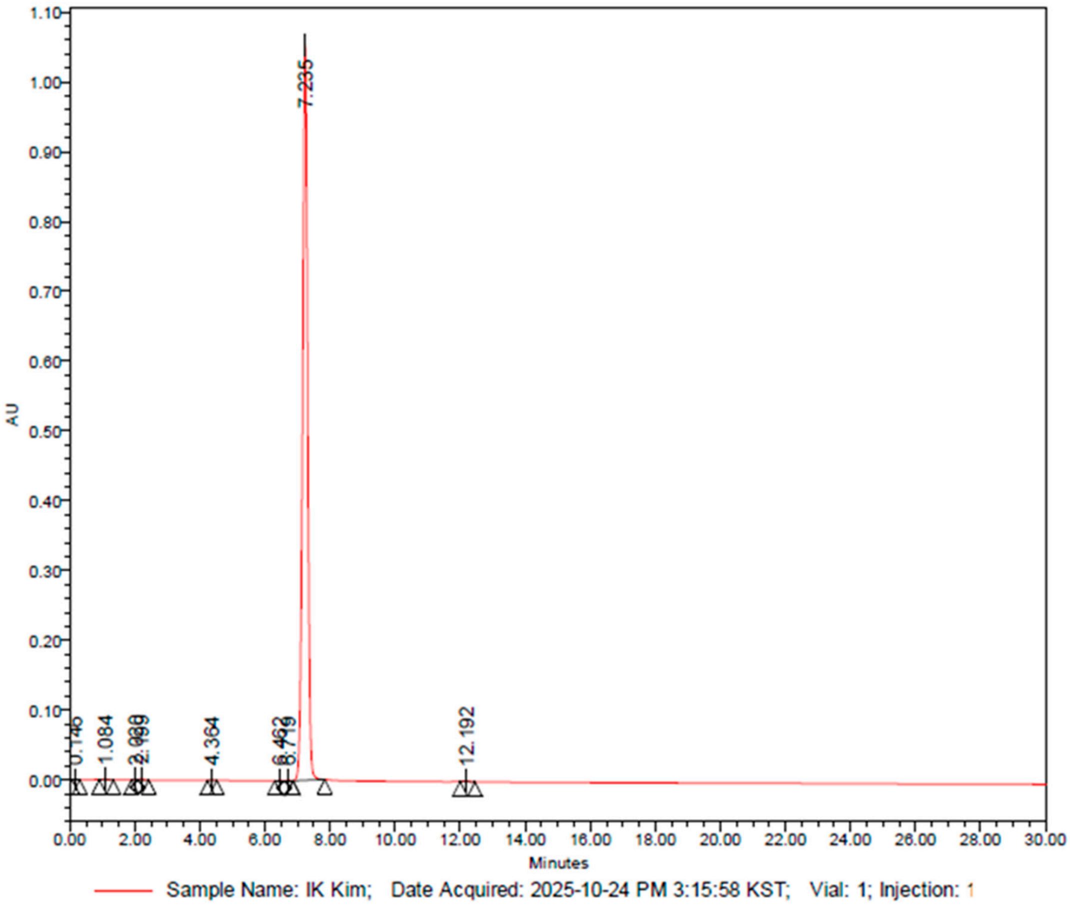

Peak Summary with Statistics

Name:

	Sample Name	Vial	Inj	Retention Time (min)	Area	% Area	Height
1	IK Kim	1	1	0.146	2896	0.03	508
2	IK Kim	1	1	1.084	9504	0.08	725
3	IK Kim	1	1	2.020	5297	0.05	1155
4	IK Kim	1	1	2.199	15260	0.13	2431
5	IK Kim	1	1	12.192	10801	0.10	783
6	IK Kim	1	1	6.462	6222	0.06	589
7	IK Kim	1	1	6.719	10409	0.09	1225
8	IK Kim	1	1	7.235	11239633	99.42	1053641
9	IK Kim	1	1	4.364	4719	0.04	649
Mean				4.714			
Std. Dev.				3.826			
% RSD				81.17			

HPLC condition	
Column	Synergi™ 4 μm, Fusion-RP 80 Å, LC Column 150 x 4.6 mm
Mobile phase	Acetonitrile : Water = 7 : 3
Flow rate	0.5 mL/min
Injection volume	10μl
Detection	254nm
Column Temperature	25 °C
Auto-sampler temperature	25 °C
Retention time	7.235 minutes

Supplementary Figure S4. Cytotoxicity test of Compound #765 in lung fibroblast.

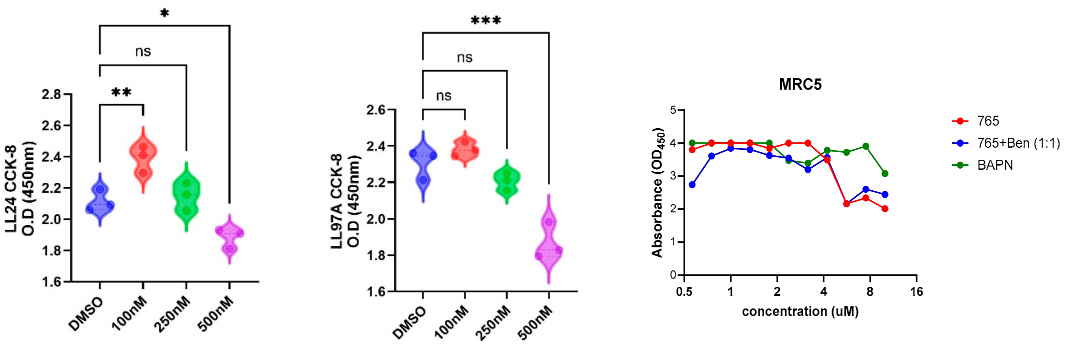

Cell viability was assessed using the CCK-8 assay in LL24, LL97A, and MRC5 cells. LL24 and LL97A treated with Compound #765 (100, 250, or 500 nM) for 72 h. No significant cytotoxicity was observed at concentrations ≤250 nM. MRC5 cell viability upon treatment with Compound #765, Compound #765 plus β-aminopropionitrile (BAPN) (1:1), or BAPN alone across a dose range (0.5–10 μM), showing minimal cytotoxic effects. Data are presented as mean ± SD (n = 3). *$p < 0.05$, **$p < 0.01$, ***$p < 0.001$; ns, not significant.

Supplementary Figure S5. Histological evaluation of preventive antifibrotic effects of Compound #765 in the bleomycin-induced pulmonary fibrosis model.

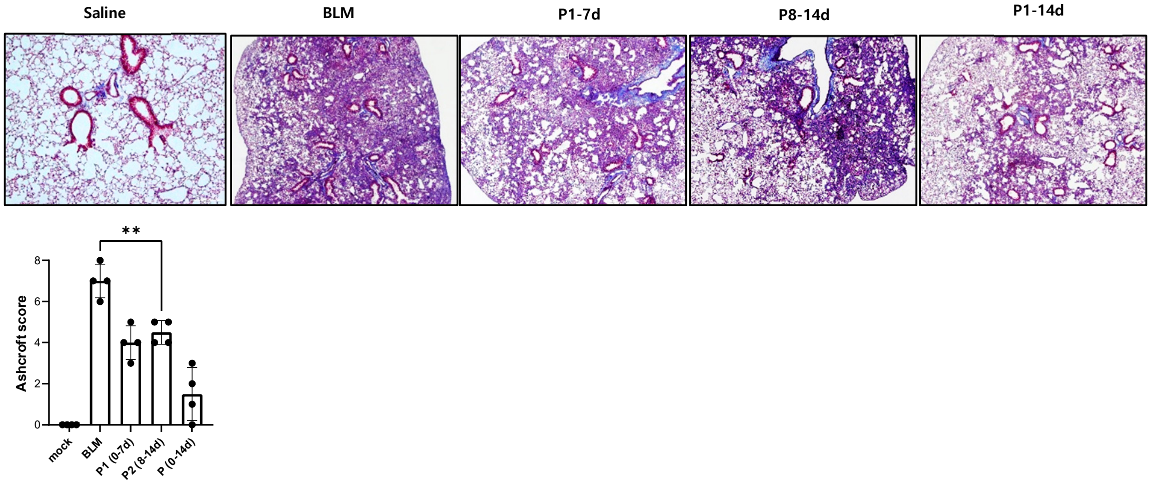

C57BL/6 mice were administered bleomycin (BLM, 1.5 U/kg, intratracheally) to induce pulmonary fibrosis. Compound #765 was administered prophylactically at 10 mg/kg under different treatment schedules. Lung tissue sections were stained with Masson's trichrome (MT) to assess collagen deposition.

Saline: No BLM; normal lung architecture with minimal collagen.

BLM: BLM-only group showing severe pulmonary fibrosis with dense collagen accumulation and alveolar wall thickening. **P1–7d**: Compound #765 administered from day 1 to 7 post-BLM (early prophylactic treatment), showing moderate reduction of collagen deposition. **P8–14d**: Treatment from day 8 to 14 post-BLM (late phase), showing partial reduction of established fibrosis. **P1–14d**: Continuous administration from days 1 to 14 showing the most prominent reduction in collagen deposition and preservation of lung structure. Ashcroft fibrosis scores for each group quantify the degree of fibrosis observed in MT-stained sections. The P1–14d group shows the most significant reduction compared to BLM-only mice. Drug preparation: Unlike the main experiments, Compound #765 was not dissolved in DMSO in this model. Instead, it was suspended in peanut oil at 10 mg/kg, then sonicated for 30 min immediately before use to ensure complete dispersion. All MT images were acquired at 40× magnification. Data are presented as mean ± SD ($n = 4–5$). $^{*}p < 0.05$, $^{**}p < 0.01$ vs. BLM group.
